# Clearance of blood stream infections in patients receiving extracorporeal membrane oxygenation: a retrospective single-center cohort study

**DOI:** 10.1186/s12879-023-08021-5

**Published:** 2023-02-02

**Authors:** Stone A. Frankford, Michal J. Sobieszczyk, Ana E. Markelz, Joseph E. Marcus

**Affiliations:** 1 Internal Medicine Residency, San Antonio Uniformed Services Health Education Consortium, JBSA-Fort Sam Houston, 3551 Roger Brooke Dr, San Antonio, TX 78234 USA; 2 Department of Medicine, Brooke Army Medical Center, JBSA-Fort Sam Houston, 3551 Roger Brooke Dr, San Antonio, TX 78234 USA; 3 Department of Medicine, Uniformed Services University, Bethesda, MD USA

**Keywords:** ECMO, Blood stream infection, Clearance

## Abstract

**Background:**

There are limited data on the treatment of blood stream infections (BSIs) in patients receiving extracorporeal membrane oxygenation (ECMO). Current guidance recommends documenting clearance only in fungal and Gram-positive BSIs. This study investigates the incidence and clinical significance of blood stream infections with positive repeat cultures (BSIPRC) in ECMO as well as clinical factors that may predict positive repeat cultures.

**Methods:**

All BSIs in patients receiving ECMO at Brooke Army Medical Center between September 2012 and October 2021 were included in this study. BSIPRC was defined as re-isolation of the same organism on repeat blood cultures following an initial positive blood culture.

**Results:**

A total of 60 patients developed 87 BSI (38.5 BSI per 1000 ECMO days). Of the 80 (92%) BSIs who had repeat blood cultures drawn, patients had BSIPRC in 35 (44%) of cases. Fever, leukocytosis, and vasopressor requirement on day of repeat culture were not associated with persistent positivity. There was no difference in survival to discharge for patients with BSIPRC as compared to single day BSI (58% vs. 63%, p = 0.78). 19% of patients with Gram-negative bacteremia had BSIPRC, and gram-negative bacteremia in general was associated with an 83% morality.

**Conclusions:**

There were no clinical findings that differentiated patients with BSIPRC from those who had a single day of positivity. BSI was associated with high mortality in patients with Gram-negative bacteremia. Given high incidence of positive repeat cultures being seen in Gram-negative BSIs, repeat blood cultures have utility for all BSIs in patients receiving ECMO.

## Background

Extracorporeal Membrane Oxygenation (ECMO) use has expanded significantly over the past decade with recent increases in veno-venous ECMO as a salvage therapy for those with reversible COVID-19 related respiratory failure or as a bridge to transplant. While ECMO has been shown to be a cost-effective tool in patients with respiratory failure, due to the critically ill nature of the patients and the need for long-term invasive access, ECMO has a high risk of hospital acquired infections [1, 2]. Blood stream infections (BSI) are associated with a three-fold increase in mortality in patients requiring ECMO support [3]. Despite the growing use of ECMO and its associated risk of infections, there are no guidelines and few data published that address the management of BSIs in ECMO patients.

One aspect of care that has limited data is the utility of repeat blood cultures for patients with BSI. For all patients, regardless of their status on an ECMO circuit, repeat blood cultures are generally recommended to document clearance in both Gram-positive and fungal BSIs [4, 5]. However, the low utility of follow-up blood cultures in Gram-negative BSI across a variety of settings have led to the practice of not testing for clearance in Gram-negative BSI. There is concern of risk for persistent BSI in critically ill patients receiving ECMO who have large cannulas in place for significant periods of time. This study aims to investigate the usefulness of repeat blood cultures in patients with BSI receiving ECMO to determine their clinical utility.

## Methods

### Study population

Positive blood cultures were reviewed from all adult patients who received ECMO at Brooke Army Medical Center, a 450-bed tertiary care center, between September 2012 and October 2021. Patients were determined to have a BSI if they had a positive blood culture and were treated with antibiotics by the treatment team. Patients were described as having blood stream infections with positive repeat cultures (BSIPRC) if an organism was re-isolated from blood cultures within 5 days of the original blood culture. At this center there is no routine antibiotic prophylaxis or use of surveillance cultures. Additionally, there was no standardized decontamination practices to include guidance on patient bathing or disinfection of the exposed ECMO circuit. Antibiotics were utilized at the discretion of the primary team without any standardized protocols related to patients receiving ECMO. The San Antonio Institutional Review Board reviewed the protocol and determined it was exempt and informed consent was not required.

### Data collection and analysis

From the medical records, variables collected in this study included: patient age and sex, ECMO indication, cannulation and decannulation dates, culture data, as well as vital signs, laboratory data, and antimicrobial therapy on day of repeat cultures. Days until clearance was defined as days between the first positive culture and the first negative culture obtained. Patients were classified as being on appropriate antimicrobials if the isolate was susceptible to the antimicrobial therapy used on the day the culture was drawn. Bacterial isolates were determined to be multi-drug resistant organisms if they were resistant to three or more classes of antibiotics, as previously defined [6].

Patients with only a single day of positive cultures were compared to BSIPRC by type of organism, use of appropriate antibiotics, and patient variables on day of repeat cultures. Mortality was compared between BSI episodes only if the isolate was the last BSI to occur in a patient’s ECMO course. Nominal variables were compared by Chi-squared or Fisher’s Exact test as appropriate. Continuous variables were compared by a Wilcoxon Rank Sum Test. A 2-sided P < 0.05 was considered statistically significant.

## Results

During the study period, 282 patients received ECMO with 60 (21%) patients developing 87 BSI (1.45 BSI per patient; 19.7 infections per 1000 ECMO days) (Table 1). This cohort was predominantly male (77%) and had a median age of 42 (IQR: 30–48). COVID-19 accounted for the majority (53%) of admissions. Patients received ECMO for a median 7.8 [IQR: 3.6–17.6] days. Gram-positive organisms accounted for the majority of BSI *with Enterococcus faecalis* (25%) *and Staphylococcus aureus* (20%) being the most commonly isolated organisms. Multi-drug resistant organisms (MDRO) were responsible for 22/87 (25%) of blood stream infections. Of the 87 BSI, 80 (92%) had at least one repeat blood culture. Of the 7 patients who did not receive a repeat blood culture, 5 (71%) died within 2 days after the initial blood culture was collected. 85/87 of the BSIs were in the setting of veno-venous ECMO, whereas two were in patients receiving veno-arterial ECMO. For patients with repeat cultures, the median duration between initial blood cultures and first repeat blood cultures was 2 days. The median days of BSI culture positivity was 4 days [IQR: 3–7] for Gram-positive infections, 3 days [IQR: 2.5–3.5] for Gram-negative infections, and 3 days [IQR: 2–3] for fungal infections.

**Table 1:** All patient and blood stream infection characteristics

Demographic factors	
Age in years, *median (IQR)*	42 (30–48)
Male, *n (%)*	46 (77%)
Hours on ECMO, *median (IQR)*	537 (337–1124)
Venovenous configuration, n (%)	85 (98%)
ECMO Indication, *n (%)*	
COVID-19	32 (53%)
Non-COVID-19 Pneumonia	10 (17%)
Burn Injury	6 (10%)
Interstitial Lung Disease	2 (3%)
Cardiomyopathy	2 (3%)
Vasculitis	2 (3%)
Other	6 (10%)
Types of Blood Stream Infection, *n (%)*	
Gram-positive	52 (60%)
Gram-negative	22 (25%)
Fungal	13 (15%)
Organisms Isolated, *n (%)*	
*Enterococcus faecalis*	22 (25%)
*Staphylococcus aureus*	17 (20%)
*Staphylococcus epidermidis*	8 (9%)
*Pseudomonas aeruginosa*	7 (8%)
*Candida albicans*	5 (6%)

Of the 80 patients with repeat blood cultures within 5 days of first positive blood culture, 35 (44%) met criteria for BSIPRC (Table 2). BSIPRC was more common in Gram-positive (48%) and fungal (45%) infections compared to Gram-negative infections (19%). There was no association between BSIPRC and maximum temperature (median 37.5 IQR [37.1–37.9] vs 37.5 [37.2–38.1], p = 0.62) or leukocyte count (15.9 [11.6–21.2] vs. 14.7 [10.2–19.7], p = 0.5) on day of repeat blood cultures. The use of initial appropriate antimicrobials was similar between BSIPRC and patients with a single day of culture positivity (71% vs. 87%, p = 0.25). Furthermore, there was no association between a patient’s additional treatment modalities such as use of vasopressors (54% vs. 49%, p = 0.65), renal replacement therapy (46% vs 44%, p = 1.0), or intubation (71% vs 62%, p = 0.47) and having a BSIPRC. Finally, there was no difference in mortality (42% vs. 37%, p = 0.78) seen in patients whose last BSI was a BSIPRC.

**Table 2:** Multiple day vs single day blood stream infection characteristics

	Multiple day (n = 35)	Single day (n = 45)	p-value^a^
Demographic factors			
Age in years, *median (IQR)*	42 (31–48)	41 (29–47)	0.53
Male, *n (%)*	26 (74%)	38 (84%)	0.28
Days between BSI and ECMO cannulation, *median (IQR)*	12 (2–52)	12 (3–43)	0.97
Types of blood stream infection, *n (%)*			
Gram-positive	24 (48%)	26 (52%)	0.35
Gram-negative	6 (19%)	13 (81%)	**0.007**
Fungal	5 (45%)	6 (55%)	1.0
Clinical factors			
On appropriate antimicrobials day of repeat culture, *n (%)*	25 (71%)	39 (87%)	0.25
Max Temperature, *median (IQR)*	37.5 (37.1–37.9)	37.5 (37.2–38.1)	0.62
Leukocyte Count, *median (IQR)*	15.9 (11.6–21.2)	14.7 (10.2–19.7)	0.50
Vasopressor Requirement, *n (%)*	19 (54%)	22 (49%)	0.65
Receiving CRRT, *n (%)*	16 (46%)	20 (44%)	1
Intubated, *n (%)*	25 (71%)	28 (62%)	0.47
Mortality, *n (%****)***	10/24 (42%)^b^	11/30 (37%)^b^	0.78

^a^Nominal variable compared by Fisher’s Exact test. Continuous variables compared by Wilcoxon Rank Sum Test

^b^Last blood stream infection of patient only assessed

In patients with BSI with Gram-negative infections, 84% died before discharge, as compared to 24% in Gram-positive infections and 38% in fungal infections. Gram-negative BSIs were more often seen in patients receiving vasopressors (74% vs. 44%, p = 0.03) and renal replacement therapy (63% vs. 39%, p = 0.11) as compared to Gram-positive and fungal infections (Table 3).

**Table 3:** Gram-negative vs gram-positive and fungal infection characteristics

	GN infections (n = 19)	GP + fungal infections (n = 61)	p-value^a^
Demographic factors			
Age in years, *median (IQR)*	39 (28–48)	40 (30–48)	0.65
Male, *n (%)*	18 (95%)	46 (75%)	0.10
Days between BSI and ECMO cannulation, *median (IQR)*	29.3 (6.0–53.5)	21.7 (3.0–45.4)	0.14
Clinical factors			
On appropriate antimicrobials day of repeat culture, *n (%)*	15 (79%)	49 (80%)	1.0
Max Temperature, *median (IQR)*	37.6 (37.1–38.1)	37.6 (37.1–38.1)	0.58
Leukocyte Count, *median (IQR)*	16.9 (13.7–20.1)	15.9 (10.3–20.3)	0.54
Vasopressor Requirement, *n (%)*	14 (74%)	27 (44%)	**0.03**
Receiving CRRT, *n (%)*	12 (63%)	24 (39%)	0.11
Intubated, *n (%)*	14 (74%)	39 (64%)	0.58
Mortality, *n (%)*	16/19 (84%)^b^	11/41 (27%)^b^	**0.0001**

^a^Nominal variables compared by Fisher’s Exact test. Continuous variables compared by Wilcoxon Rank Sum Test

^b^Last blood stream infection of patient only assessed

The subset of patients who developed Gram-negative BSIPRC were examined (Table 4). Most Gram-negative BSIPRC occurred in patients with COVID-19 and did not involve multi-drug resistant isolates. The median duration of positive blood cultures was five days and five (83%) of the patients died, two of which who never demonstrated clearance. Interestingly, both cases occurred in patients with *Pseudomonas aeruginosa* with one patient having had bacteremia for 28 days as well as isolation from sputum cultures.

**Table 4:** Gram-negative blood stream infections with positive repeat cultures

Organism	Days of positive cultures	Days until clearance	Admission diagnosis	MDR	Appropriate antibiotics	Survival to discharge
*E. anophelis*	5	6	COVID-19	No	No	No
*E. cloacae*	3	4	Thermal Burn	No	Yes	Yes
*K. oxytoca*	11	13	COVID-19	No	Yes	No
*P. aeruginosa*	2	N/A^a^	COVID-19	No	No	No
*P. aeruginosa*	28	N/A^a^	COVID-19	No	Yes	No
*P. rettgeri*	5	7	COVID-19	Yes	No	No

^a^Patient died before clearance

## Discussion

Our study is the first to describe BSIPRC in patients requiring ECMO. Our findings show that Gram-positive and fungal infections have BSIPRC more frequently than Gram-negative infections without clear clinical criteria of what patients are likely to have repeat positivity. We also found that BSIPRC is associated with high mortality in Gram-negative infections. This study suggests a possible benefit of obtaining repeat blood cultures in patients receiving ECMO for all BSI regardless of infective organism.

The prevalence of ECMO BSI infections in the pre-COVID era has been reported around 5.5–18% [7–11]. Data looking at BSI’s in those specifically on ECMO due to SARS-CoV-2 infections is scarce. However several single center retrospective studies have reported BSI infection rates as high as 32–48% [12, 13]. Our reported BSI prevalence of 21% reflects a population with mixed indications for ECMO, however when specifically looking at the 70 patients in our study who were placed on ECMO secondary to SARS-CoV-2, we see the prevalence of BSI is 46%. Proposed explanations for this observation include higher rates of central line-associated bloodstream infections (CLABSI) reported during the COVID-19 pandemic [14], high rates of pre-cannulation blood stream infections (12%), bacterial pneumonia co-infection (33%) seen in COVID-19 patients prior to ECMO initiation [15], and higher rates of secondary infections, especially VAP in COVID-19 patients post-cannulation, when compared to influenza controls [16, 17].

ECMO is associated with a high rate of secondary infections [18]. There are many proposed explanations for the high infection rate in ECMO including a predisposition to renal failure resulting in immunosuppression and dysregulation of the coagulation system, which promotes bacterial adhesion to catheters, or sequestration of leukocytes by the circuit [2, 16, 18–24]. Other possible etiologies include colonization of the ECMO catheter or membrane oxygenator [25, 26]. All of these mechanisms could contribute to the high number of patients with BSIPRC.

The organisms seen in this cohort are similar to the diversity previously described. Interestingly there was a high rate of *Enterococcus faecalis* as compared to previous studies. Nationally, *E. faecalis* is associated with nosocomial infections causing 7.7% of CRBSIs [27]. Enterococcal bacteremia has been shown to correlate with prolonged ICU stays [28]. The mean duration of ECMO hours in previous ECMO studies ranged from 168 to 307 [8, 29, 30] whereas in this study, the median duration of ECMO hours was 537 h and the longer time on ECMO may be a reason for increased infection rates. Further studies are needed to better elucidate the causes of bacteremia in ECMO.

While it is recommended practice to collect repeat blood cultures in patients who have Gram-positive or fungal BSIs, this is not the case for Gram-negative BSI [4, 5]. Previous large studies of Gram-negative bacteremia in hospitalized patients have shown that positive repeat cultures in the setting of gram-negative BSI are seen 6–11% of the time [31, 32]. When specifically looking at a subset of immunocompromised hospitalized patients, the frequency of positive repeat cultures in gram negative BSIs continues to be comparatively low at 3% [33]. At one academic center, BSIPRC was only seen in 4/38 of critically ill patients and was more commonly seen with endovascular sources of infection [34]. This low prevalence differs greatly from our study where 19% of patients with Gram-negative bacteremia had a positive repeat culture, which is similar to previously reported studies for Gram-positive organisms [31]. While in our study, we only observed patients with repeat cultures, a large metanalysis suggest mortality benefit for ordering repeat cultures in Gram-negative bacteremia [35]. Given the increased incidence of Gram-negative BSIPRC observed in this cohort, it would be reasonable to obtain repeat cultures for all BSIs in those receiving ECMO.

Furthermore, in this small study, there was no clinical criteria that differentiated patients who had BSIPRC from those who had single isolation of a pathogen. In studies limited to bacteremia, case–control analyses have also not shown differences in fever or leukocytosis between those persistently bacteremic [32]. This inability to identify patients with persistent BSI is even more plausible in a system such as ECMO where various vital signs such as temperature and blood pressure can be partially controlled by the circuit. Without reliable clinical signs that would suggest a BSIPRC, there is a further reason for repeating cultures to demonstrate clearance as the utility of clinical parameters do not appear to correlate.

In our cohort, mortality amongst those with Gram-negative bacteremia was high (83%), especially in those with BSIPRC, where 5/6 patients died before discharge. Few were multidrug resistant and half were on appropriate antibiotics at time of repeat blood cultures. There were two patients with significantly prolonged bacteremia despite appropriate antibiotics after their repeat culture was obtained. In the setting of proper antibiotics being used, this argues for a lack of source control. One possibility of a deep source is the lungs. In ECMO studies of ventilator-associated pneumonia (VAP), recurrence culture positivity has been described as high as 79%, suggesting possible seeding from a protected space such as fibrotic lungs [16]. Additionally, previous studies have demonstrated gut hypoperfusion leading to increased gut permeability in those on cardiopulmonary bypass, further suggesting that a pathogenically colonized GI tract serves as another plausible source [36, 37].

This single center, retrospective, observational study is subject to several limitations. All cultures acquired by clinical team as part of clinical care without standardized protocols for repeat cultures. There was no standard antimicrobial regimen and therefore, only 80% of patients were on appropriate antibiotics at time of repeat culture. As primary teams were the ones who differentiated real infections from contaminants, it is possible that there were contaminants that were included in analysis. A majority of patients in this study were infected with SARS-CoV-2, which may differ from centers that treat different patient populations. It is unclear if the high mortality rate observed in those who developed Gram-negative BSIs is from the infection itself or rather was a manifestation of a critically ill patient. Finally, best practices have not yet been established for cannula or circuit exchange in the setting of bacteremia and will need further studies to better characterize.

## Conclusion

In conclusion, this study evaluated BSIs in patients receiving ECMO and found that BSIPRC was commonly seen in Gram-positive and fungal infections, but were also seen in Gram-negative infections at much higher rates than described in patients who are not receiving ECMO. BSIPRC is associated with a high mortality in Gram-negative infections and there is no clinical data point to differentiate patients with a single day of positive cultures from a patient with multiple days of positive cultures. Therefore, in ECMO, it is reasonable to get repeat blood cultures in all patients with a BSI, regardless of the pathogen isolated.

## Data Availability

The datasets used and/or analyzed during the current study are available from the corresponding author on reasonable request.

## Abbreviations

BSI: Blood stream infections
ECMO: Extracorporeal membrane oxygenation
BSIPRC: Blood stream infections with positive repeat cultures
MRDO: Multi-drug resistant organism
CLABSI: Central line-associated bloodstream infections
VAP: Ventilator-associated pneumonia
